# Clinical Outcomes for Patients With Ulcerative Colitis in Cases of Withdrawal and Resumption of Janus Kinase Inhibitors: Multicenter Cohort Study

**DOI:** 10.1093/crocol/otaf020

**Published:** 2025-03-22

**Authors:** Yasuki Sano, Yuka Ito, Naoto Yagi, Yusuke Honzawa, Norimasa Fukata, Makoto Naganuma

**Affiliations:** Third Department of Internal Medicine, Division of Gastroenterology and Hepatology, Kansai Medical University, Osaka, Japan; Third Department of Internal Medicine, Division of Gastroenterology and Hepatology, Kansai Medical University, Osaka, Japan; Third Department of Internal Medicine, Division of Gastroenterology and Hepatology, Kansai Medical University, Osaka, Japan; Third Department of Internal Medicine, Division of Gastroenterology and Hepatology, Kansai Medical University, Osaka, Japan; Third Department of Internal Medicine, Division of Gastroenterology and Hepatology, Kansai Medical University, Osaka, Japan; Third Department of Internal Medicine, Division of Gastroenterology and Hepatology, Kansai Medical University, Osaka, Japan

**Keywords:** ulcerative colitis, JAK inhibitors, treatment discontinuation, remission, relapse

## Abstract

**Background:**

Janus kinase inhibitors (JAKis) have revolutionized ulcerative colitis (UC) management; however, the consequences of treatment discontinuation in patients achieving clinical remission remain poorly understood. This study investigated the clinical outcomes following JAKi discontinuation and retreatment effectiveness in patients with relapse.

**Methods:**

In this multicenter retrospective cohort study, we analyzed 101 patients with UC who received their first JAKi treatment between 2018 and 2024. Among them, 53 who achieved remission (Patient-Reported Outcome 2 = 0) in week 8 were included. The primary endpoint was a comparison of relapse-free survival between the treatment continuation and discontinuation groups (n = 37 and 16, respectively). The secondary endpoints included assessment of post-discontinuation remission maintenance and post-retreatment remission rates.

**Results:**

The proportion of female patients in the discontinuation group (68.8%) was higher (P = .0478) than the continuation group (40.5%). The mean relapse-free survival was significantly longer in the continuation group than in the discontinuation group (1679 vs 882 days, cumulative relapse-free rate 83.3% vs 13.6%, P < .001, respectively). In the latter, 13 patients experienced relapse during follow-up (post-discontinuation mean relapse-free survival: 326 days), although all patients remained in clinical and biological remission. Notably, among patients who received JAKi retreatment, 83.3% achieved remission in week 8.

**Conclusions:**

To our knowledge, this is the first real-world study to evaluate the effects of JAKi discontinuation on the outcomes for patients with UC. JAKi discontinuation in patients in remission was associated with a high relapse risk. JAKi retreatment was highly effective in patients who experienced relapse after treatment discontinuation, providing valuable evidence for managing treatment interruption.

## Graphical Abstract

**Graphical Abstract:**
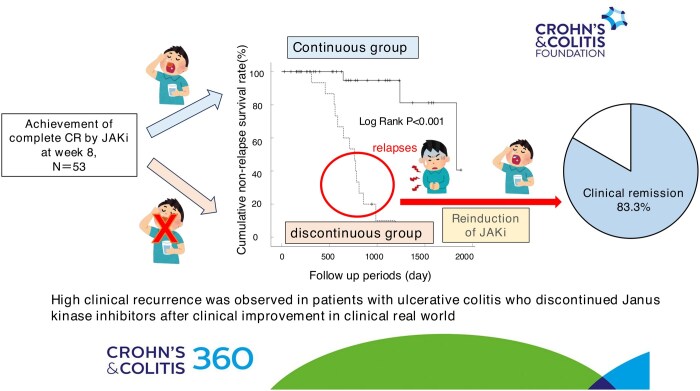

## Introduction

The clinical management of ulcerative colitis (UC) has been revolutionized by the introduction of Janus kinase inhibitors (JAKis), which have shown remarkable efficacy as both induction and maintenance therapies.^1–3^ These agents represent a significant advancement in the treatment of refractory UC and offer an oral treatment alternative to traditional biologics. However, the increasing clinical experience with JAKis has raised important considerations regarding their long-term use and safety profile.

Recently, safety signals have emerged from studies on other inflammatory conditions. A pivotal trial in patients with rheumatoid arthritis highlighted the increased risks associated with long-term tofacitinib (TOFA) use compared with tumor necrosis factor inhibitors, including higher incidences of major adverse cardiovascular events (MACEs), malignancies, opportunistic infections, herpes zoster, and non-melanoma skin cancers over a median 4-year follow-up period.^4^ While a subsequent study specifically examining patients with inflammatory bowel disease (IBD) showed no elevated risks of both MACEs and malignancies associated with JAKi use,^5^ these findings underscore the necessity for careful risk-benefit assessments in long-term therapy.

In addition to safety considerations, several practical challenges complicate long-term JAKi maintenance therapy. These include the potential teratogenic effects in women of childbearing age, substantial treatment costs, and varying patient preferences regarding chronic medication use. Such factors frequently necessitate the consideration of JAKi discontinuation in clinical practice.

The importance of maintenance therapy has been well demonstrated in biological treatments, as evidenced by the HAYABUSA study, which showed significantly higher relapse rates at 48 weeks in patients who discontinued infliximab than in those who continued treatment.^6^ Similarly, previous pivotal studies on JAKis have consistently reported lower clinical remission (CR) rates for a year in placebo groups than in active treatment groups,^1–3^ suggesting that JAKi discontinuation might lead to a high risk of relapse in real-world settings as well. However, the consequences of JAKi withdrawal in patients with UC who have achieved CR remain poorly understood in a clinical setting, creating uncertainty for both clinicians and patients when faced with decisions about treatment discontinuation.

This study addresses 2 critical knowledge gaps regarding JAKi treatment for UC. The primary objective of this study was to investigate the relationship between JAKi discontinuation and long-term clinical outcomes after the achievement of CR with JAKi therapy. Additionally, the effectiveness of JAKi retreatment in patients who experienced disease relapse following treatment discontinuation was evaluated. We believe these findings will provide essential guidance for clinicians managing patients with UC on JAKi treatment and inform evidence-based decisions regarding treatment withdrawal.

## Materials and Methods

### Study Design and Patient Selection

This multicenter retrospective cohort study was conducted among patients with UC who were treated at 3 hospitals. Between 2018 and 2024, 101 patients with UC received their first JAKi treatment with TOFA, filgotinib (FIL), or upadacitinib (UPA). The diagnosis of UC was confirmed using established clinical, radiological, endoscopic, and pathological criteria.^7^

Patients with low disease activity (defined as a Patient-Reported Outcome 2 [PRO2] score of 0 or 1) at the time of JAKi initiation were excluded. Among the patients who achieved a clinical response in week 8, those who discontinued treatment owing to adverse events or failed to achieve CR during the observation period were excluded. Based on the Selecting Therapeutic Targets in Inflammatory Bowel Disease II criteria,^8^ clinical response was defined as a decrease of at least 50% in the PRO2 score (based on rectal bleeding and stool frequency), whereas CR was defined as a PRO2 score of 0. Clinical relapse was defined as the worsening of clinical symptoms or endoscopic mucosal inflammation requiring additional treatment or a change in treatment, including escalation of the oral 5-aminosalicylic acid (5-ASA) dosage, addition of 5-ASA or corticosteroid enemas or suppositories, or initiation of new therapeutic agents (see Figure, Supplementary Data Content 1).

### Data Collection

Data were extracted from the medical records of both outpatient and inpatient patients with UC. Follow-up data, including laboratory test results, endoscopic findings, and those pertaining to outpatient visits, were collected through a systematic review of electronic medical records. Demographic data, disease characteristics, previous treatments, and clinical outcomes were collected from patients’ medical charts. Laboratory test data, including hemoglobin, albumin, and C-reactive protein levels, were collected in week 8. Regarding comedication use, data on the use of corticosteroids, thiopurines, and biologics were collected before JAKi initiation. Biologic use was defined as the use of infliximab, adalimumab, golimumab, vedolizumab, ustekinumab, mirikizumab, and risankizumab, which were permitted by the Japanese health insurance system .

### Study Groups and Endpoints

Patients were categorized into 3 groups: those who continued JAKi treatment at the primary dose without reduction, those who continued JAKi treatment with dose reduction, and those who discontinued JAKi treatment after achieving CR. The primary endpoint was the comparison of relapse-free survival from the time of JAKi initiation between the continuation and discontinuation groups by using Kaplan-Meier analysis and the log-rank test. For the sensitivity analysis, relapse-free survival from the time of TOFA was also evaluated between the continuation and discontinuation groups. The secondary endpoints were as follows: (1) to compare clinical characteristics between the continuation and discontinuation groups; (2) to assess the remission maintenance rate after JAKi discontinuation by using Kaplan-Meier analysis, with day 0 defined as the day of JAKi discontinuation; and (3) to assess clinical outcomes after JAKi retreatment in patients who relapsed following treatment discontinuation.

### Approach to Potential Biases

To minimize the measurement bias, 2 approaches were implemented: (1) systematic collection of patient symptoms through standardized questionnaires completed by patients at each clinical visit and (2) recording of PRO2 scores by treating physicians during the same visits to ensure consistency in evaluation. This dual assessment approach was aimed at systematically capturing both patient-reported outcomes and physician evaluations.

### Statistical Analysis

Missing data were handled as follows. For PRO2 scores, only patients with complete data at baseline and week 8 were included in the analysis. Laboratory test data missing at week 8 were substituted with the closest available data within ± 1 week. Patients were censored at their last follow-up visit if they were lost to follow-up or reached the end of the observation period without experiencing a relapse. Continuous variables are reported as median values (interquartile range) and compared using the Mann-Whitney *U* test. Categorical variables are expressed as percentages and compared using Fisher’s exact test or the chi-square test, as appropriate. Kaplan-Meier curves were generated to depict the cumulative relapse-free survival rate, and differences between the groups were compared using the log-rank test. Statistical significance was set at *P* < .05. All statistical analyses were performed using SPSS version 26 (IBM Corp.).

### Ethical Considerations

The study was conducted in accordance with the Declaration of Helsinki and the Ethical Guidelines for Medical and Health Research Involving Human Subjects. The study design was reviewed and approved by the Ethics Committee of our institution. An opt-out approach was used for consent because this was a retrospective observational study and there was no risk to the participants. Patients were allowed to refuse participation in this study by posting their preferences on the institutional website (http://www.kmu.ac.jp/hirakata/hospital/2671t800000135zj.html.). If the patients were younger than 20 years of age, their parents or guardians also had the right to refuse.

## Results

### Patient Disposition and Baseline Characteristics

In total, 101 patients received their first JAKi treatment (TOFA: *n* = 59; FIL: *n* = 24; and UPA: *n* = 18). Among them, 13 patients with mild disease activity at baseline (PRO2 score 0-1) were excluded. Furthermore, 23 patients were excluded because of either a lack of a clinical response within 8 weeks or treatment modifications necessitated by adverse events.

Of the remaining 65 patients who achieved a clinical response in week 8 (TOFA: *n* = 39; FIL: *n* = 14; UPA: *n* = 12), 53 (TOFA: *n* = 32, FIL: *n* = 10, UPA: *n* = 11) achieved CR. These patients were subsequently divided into 2 groups: 37 continued treatment and 16 attempted treatment discontinuation after achieving remission (see Figure, Supplementary Data Content 1).

The clinical characteristics of the patients are listed in Table 1. The baseline characteristics and clinical status at week 8 were compared between the continuation and discontinuation groups. Demographic characteristics showed significant differences in sex distribution (male/female patients: 22/15 vs 5/11, *P* < .05) and years since diagnosis (continuation group vs discontinuation group: 5 [1-13] vs 7.5 [2-11.5], *P* < .05) between the groups. The disease extent was pancolitis, left-sided colitis, and proctitis in 22, 13, and 2 patients, respectively. The corresponding patient numbers in the discontinuation group were 10, 6, and 0. Regarding previous treatments, steroid-dependent and biological-naïve patients comprised 62.2% and 70.3% of the patients in the continuation group, respectively. The corresponding percentages in the discontinuation group were 62.5% and 75.0%. Disease activity assessed based on the PRO2 score was comparable between the groups at baseline (continuation group vs discontinuation group: 4 [3-5] vs 4 [3-5], *P* = .95) and week 8 (continuation group vs discontinuation group: 0 [0-1] vs 0.5 [0-1], *P* = .65). Laboratory test data in week 8, including hemoglobin, albumin, and C-reactive protein levels, were not significantly different between the groups. The duration of JAKi administration at the initial dose was similar between the continuation and discontinuation groups (84 [57-178] vs 70 [57-303] days, respectively, *P* = .95).

**Table 1.** Patient characteristics.

Characteristic	Continuation	Discontinuation	*P*-value
	(*N* = 37)	(*N* = 16)	
Type of JAKi used (TOFA/FIL/UPA)	17/10/10	15/0/1	
Age, median (IQR), years	42 [29-55]	33.5 [28-55]	0.7241
Sex (*n*) (male/female)	22/15	5/11	0.0476*
Years from diagnosis, median (IQR)	5 [1-13]	7.5 [2-11.5]	0.0287*
Disease extent			
Pancolitis	22	10	0.236
Left-sided colitis	13	6	0.24
Proctitis	2	0	0.483
Steroid response/history			
Dependent	23 [62.2%]	10 [62.5%]	0.242
Refractory	10 [27.0%]	6 [37.5%]	0.188
Steroids-naïve	4 [10.8%]	0	0.226
Previous use of thiopurine,(%)	17 [45.9%]	10 [62.5%]	0.39
Number of previous biological treatment, (*n*)			
0	26 [70.3%]	12 [75.0%]	0.249
1	10 [27%]	3 [18.8%]	0.232
2	1 [2.7%]	1 [6.3%]	0.43
PRO2 score at baseline, median (IQR)	4[3-5]	4 [3-5]	0.95
PRO2 score at 8 week, median (IQR)	0 [0-1]	0.5 [0-1]	0.65
Laboratory data at 8 week, median(IQR)			
Hemoglobin (g/dL)	12.8 [11.8-14.5]	13.0 [11.8-13.5]	0.98
Albumin (g/dL)	4.3 [4.0-4.5]	4.5 [4.0-4.7]	0.436
C-reactive protein (mg/mL)	0.013 [0.006-0.092]	0.013 [0.010-0.545]	0.45
Duration of time administered at the initial dose (days)	84 [57-178]	70 [57-303]	0.95
Duration of JAKi administration (days)	651.5[297-1085]	516 [335-733]	0.28

Abbreviations: FIL, filgotinib; IQR: interquartile range; PRO2: patient-reported outcome 2; TOFA, tofacitinib; UPA, upadacitinib.

### Relapse-Free Survival From the Time of JAKi Treatment Initiation in the Continuation and Discontinuation Groups

**Figure 1.**
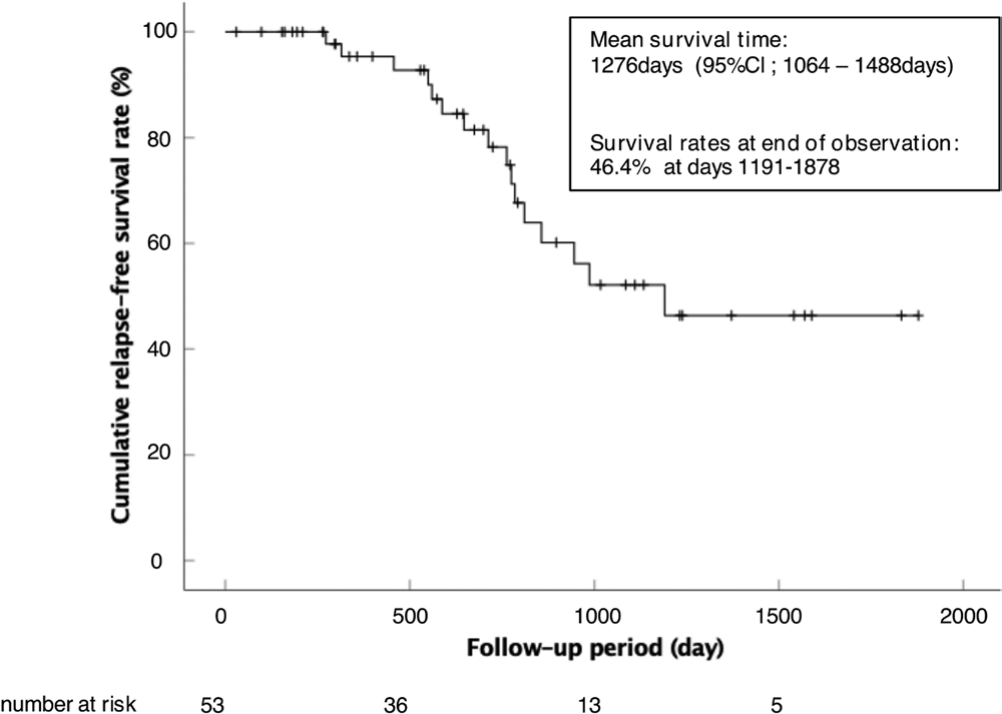
Overall rate of cumulative remission maintenance. Only patients with Patients Report Outcomes 2 of 0 at week 8 were collected as remission maintenance.

In all 53 patients who achieved CR, the mean survival time was 1276 days (95% CI, 1064-1488 days). The relapse-free survival rate at the end of the observation period was 46.4% (days 1191-1878). Kaplan-Meier survival analysis was performed to evaluate relapse rates (Figure 1).

**Figure 2.**
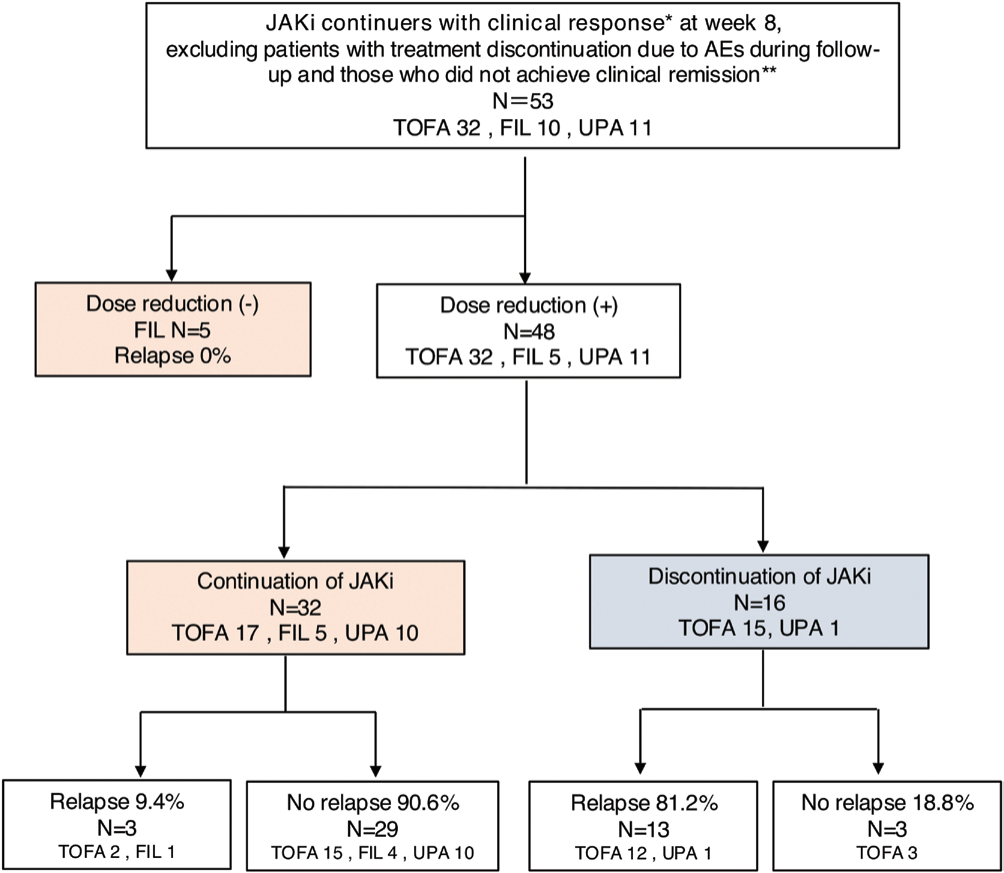
Progress in cases in which clinical remission was achieved at 8 weeks and Janus kinase inhibitors were continued.

The continuation group comprised 37 patients in total: 5 who continued the initial dose of FIL without dose reduction and 32 who continued treatment after dose reduction. The discontinuation group included 16 patients who discontinued JAKi treatment after dose reduction. During the observation period, none of the patients who continued the initial FIL dose experienced a relapse (0%), whereas 3 patients (9.4%) in the dose reduction continuation group relapsed. Although the dose of JAKi was increased in all 3 patients who had recurrence after a reduction, CRs were not obtained. Therefore, UPA was administered to 2 patients, while systemic corticosteroid was administered for remission induction in one patient, leading to CR in all patients. In contrast, 13 patients (81.2%) in the discontinuation group experienced a relapse (Figure 2).

**Figure 3.**
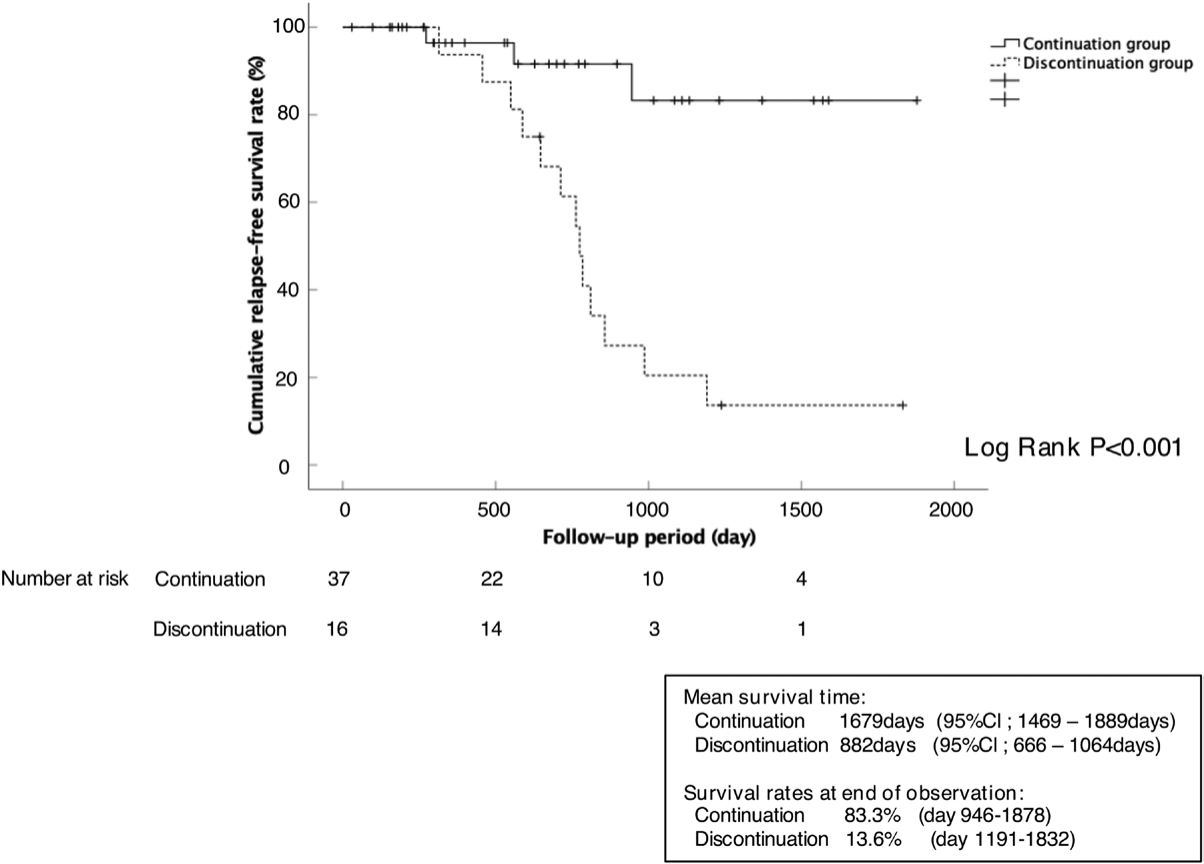
Kaplan-Meier analysis of the rate of cumulative relapse-free survival in patients in the continuation group vs the discontinuous group.

Comparison of the Kaplan-Meier curves between the continuation and discontinuation groups by using the log-rank test revealed a significant difference (*P* < .001). The mean relapse-free survival time was 1679 days (95% CI, 1469-1889 days) in the continuation group, which was substantially longer than that in the discontinuation group (882 days; 95% CI, 666-1064 days). Furthermore, the cumulative relapse-free rate was markedly higher (*P* < .001) in the continuation group (83.3%) than in the discontinuation group (13.6%) (Figure 3).

For the sensitivity analysis, only patients treated with TOFA were evaluated. Like patients in all cohort, the mean relapse-free survival time in the continuation group (1698 days; 95% CI, 1464-1931 days) was significantly longer than that in the discontinuation group (902 days; 95% CI, 675-1128 days) (*P* < .001). Furthermore, the cumulative relapse-free rate was markedly higher in the continuation group (85.2%) than in the discontinuation group (14.5%) (see Figure, Supplementary Data Content 2).

### Remission Maintenance After JAKi Discontinuation

**Figure 4.**
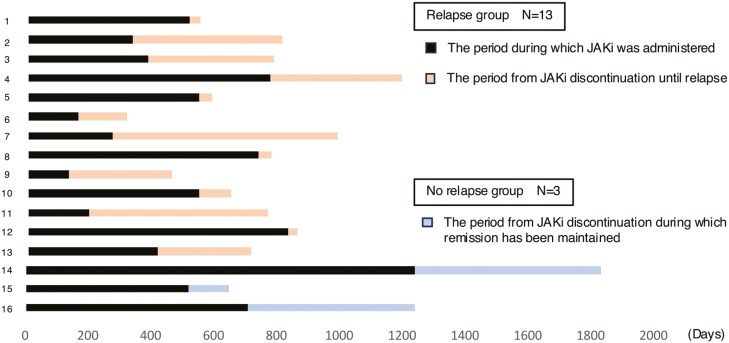
Periods of Janus kinase inhibitor (JAKi) treatment and discontinuation for each of the cases in which JAKis were discontinued.

Sixteen patients discontinued JAKi treatment after achieving CR. In all patients, JAKi was discontinued during the maintenance phase, with treatment duration ranging from 131 to 1236 days (median 516 [335–733] days) before discontinuation. Kaplan-Meier analysis, with day 0 defined as the day of JAKi discontinuation, revealed the mean survival time to be 326 days (95% CI, 133-518 days). The final relapse event occurred on day 717 when the relapse-free survival rate reached 0% (see Figure, Supplementary Data Content 3). Individual patient data visualization showed the duration of JAKi treatment before discontinuation and the subsequent time to relapse or the last follow-up for each patient (Figure 4). Among the 16 patients who discontinued JAKi treatment, 13 experienced a relapse, while 3 patients were censored on days 128, 532, and 596.

### Characteristics of Patients who Discontinued JAKi Treatment

Thirteen patients (81.3%) discontinued treatment after achieving remission through shared decision-making with their physicians, whereas 3 (18.7%) discontinued treatment without physician consultation. At the time of discontinuation, all patients had achieved CR and biological remission with a PRO2 score of 0 and had favorable laboratory parameters, including C-reactive protein (0.034 [0.01-0.17] mg/dL), albumin (4.5 [4.2-4.9] g/dL), and hemoglobin (12.9 [11.3-14.3] g/dL) levels. Furthermore, fecal immunochemical tests (FITs) revealed low disease activity (20 [2-20]) (Table 2). The decision to discontinue was influenced by several factors during the study period, including concerns about an increased infection risk during the coronavirus disease 2019 pandemic, a September 2021 safety warning by the Food and Drug Administration regarding the increased risks of serious adverse events associated with JAKi use (including major cardiovascular events and malignancy), and pregnancy planning in women of childbearing age. Some patients discontinued JAKi at their own request; we stopped JAKi for individuals with clinical and biological remissions, including these patients. Other patients discontinued JAKi because they were biologics-naïve. After JAKi discontinuation, 10 patients (62.5%) were maintained on 5-ASA monotherapy, while only 3 (18.7%) received thiopurine-containing regimens (azacitidine [AZA] monotherapy: 1, 5-ASA + AZA: 2).

**Table 2.** Clinical characteristics at the time of JAKi discontinuation and post-discontinuation treatment details (*n* = 16).

Age, median [IQR], years	33.5 [28.5-51.5]
Sex (*n*) (male/female)	5/11
Reasons for discontinuation *n* (%)	
Clinical remission	13 (81.3%)
Self-discontinuation	3 (18.7%)
Clinical parameters at discontinuation,	
PRO2 score, median [IQR]	0 [0-0]
C-reactive protein (mg/dL), median [IQR]	0.034 [0.01-0.17]
Albumin (g/dL), median [IQR]	4.5 [4.2-4.9]
Hemoglobin (g/dL), median [IQR]	12.9 [11.3-14.3]
Fecal immunochemical test score, median [IQR]	20 [2-20]
History of thiopurine use *n* (%),	
Never use	5 (31.3%)
Previous use	11 (68.7%)
Maintenance therapy after discontinuation *n* (%),	
None	3 (18.7%)
5-ASA monotherapy	10 (62.5%)
AZA monotherapy	1 (6.3%)
5-ASA + AZA	2 (12.5%)

Abbreviations: 5-ASA, 5-aminosalicylic acid; AZA, azacytidine; IQR, interquartile range; JAKi, Janus kinase inhibitor; PRO2, patient-reported outcome 2.

### Efficacy of JAKi as the Reinduction Treatment After a Relapse in the Discontinuous Group

**Figure 5.**
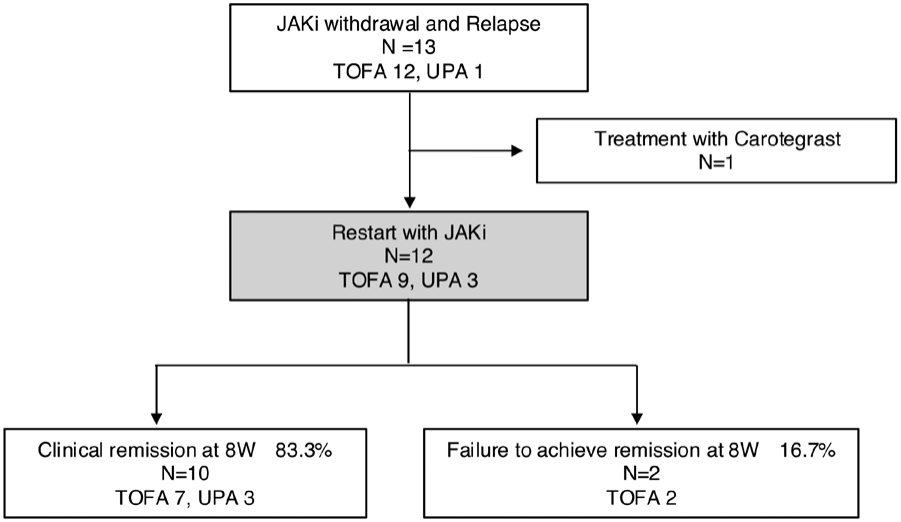
Clinical outcomes in cases of relapse after the discontinuation of Janus kinase inhibitors.

Among the 13 patients who relapsed after JAKi discontinuation, 12 received JAKi retreatment. In week 8 after retreatment, 10 patients (83.3%) achieved CR and 2 failed to achieve remission (Figure 5). Clinical characteristics seemed to be  comparable between patients who achieved CR after reinduction and those who did not, although this shoule be interupted with caution as there were only 2 cases of relapse (see Table, Supplementary Data Content 4).

## Discussion

This study showed 2 important findings regarding JAKi treatment in patients with UC who achieved CR. First, the discontinuation of JAKi treatment was associated with a high risk of relapse, even in patients who experienced excellent treatment outcomes. Although few studies have evaluated the clinical outcomes in patients who discontinued JAKi treatment in clinical settings, our study revealed that the majority of patients who discontinued JAKi treatment experienced a relapse during the follow-up period, even though CR and biological remission were achieved when JAKi treatment was discontinued, highlighting the importance of JAKis as a maintenance therapy. Importantly, we also demonstrated, for the first time to our knowledge, in a real-world setting that JAKi retreatment was highly effective in patients who experienced a relapse after treatment discontinuation. This finding is particularly significant as it provides practical evidence supporting the JAKi retreatment strategy in clinical practice, consistent with the findings from large-scale trials of TOFA and FIL.^9–11^ These results suggest that while the maintenance of JAKi treatment is crucial for sustained remission, even in cases where discontinuation leads to a relapse, JAKi retreatment can be an effective strategy for regaining disease control.

Recent evidence supports the favorable long-term safety profile of JAKi treatment. Regarding MACEs in patients with IBD, recent studies indicated that most patients receiving TOFA have a relatively low risk of MACEs, with previous cardiovascular disease being the primary risk factor.^5–12^ While concerns about herpes zoster should be raised, particularly in Asian populations,^13–16^ a recent population-based study of patients with rheumatoid arthritis or UC demonstrated that continued JAKi treatment was not associated with a marked risk of subsequent recurrent Herpes zoster reactivation.^17^ Furthermore, UPA has shown safety outcomes comparable to those of other active treatments or placebos in patients with immune diseases.^18^ Recent post hoc analyses and real-world data have consistently shown no evidence that long-term JAKi treatment considerably increases the risk of serious adverse events.^19,20^ Despite this reassuring safety profile, we found that some patients in our cohort requested the discontinuation of JAKi treatment for various reasons, including concerns during the coronavirus disease 2019 pandemic and the high cost of treatment.

Although JAKis are commonly discontinued for various reasons in clinical practice, few studies have examined the effects of JAKi discontinuation in patients with IBD in a real-world setting. We found that patients who discontinued JAKi treatment were at substantial risk of relapse even after achieving CR and biological remission. However, although large-scale trials of TOFA and FIL have demonstrated high efficacy rates in regaining remission after discontinuation,^10,11^ the effectiveness of reinduction therapy in real-world settings has not been previously investigated. Our study is the first to demonstrate that timely reinitiation of JAKi treatment is highly effective in patients who experience a relapse after treatment discontinuation in a real-world setting, with a high proportion of patients achieving CR upon JAKi retreatment. These results suggest that while patients who require JAKi treatment should ideally continue it unless there are specific reasons for discontinuation, careful monitoring, and appropriate timing of retreatment can effectively manage disease recurrence when discontinuation is necessary.

Regarding treatment discontinuation in real-world settings, our analysis revealed a significantly higher proportion of younger female patients of childbearing age in the discontinuation group, suggesting that concerns regarding the potential risks of JAKi use during pregnancy markedly influenced treatment decisions. Because the safety of these medications in pregnant patients has not yet been fully established, both patients and physicians may opt for treatment discontinuation to avoid potential risks to the fetus. A recent report on pregnancy outcomes in patients treated with UPA indicated that the rates of adverse pregnancy outcomes following UPA exposure were comparable to those observed in the general population.^21^ However, the study also showed that 21% of the cases decided on electrical termination. Some female patients who hoped to conceive tended to discontinue JAKi treatment as early as possible after achieving remission. For patients in whom JAKi discontinuation is necessary, alternative maintenance strategies should be considered during pregnancy, including the combination of 5-ASA and thiopurine drugs or switching to biological therapies with established safety profiles. However, the options for maintenance therapy were often limited in our study population. Many patients who required JAKi treatment had a history of treatment failure or intolerance to thiopurine drugs, and the optimal use of alternative maintenance therapies, including thiopurines or biologics, may not have been fully implemented in some cases. Patients who wish to discontinue JAKi tend to dislike expensive treatments and are reluctant to use other biologics. The 3 patients who discontinued JAKi against their physician’s opinions voluntarily stopped it due to medical costs, despite a special insurance system covering most of their medical expenses. These findings highlight the importance of carefully considering and maximizing all available maintenance therapy options when JAKi discontinuation is necessary.

This study had some limitations. First, we evaluated several drugs (TOFA, FIL, and UPA) while performing sensitivity analysis, and a similar result was confirmed even in patients receiving TOFA alone. Second, there was no pre-specified observation period, which could have affected the interpretation of the results. Third, the observation period for patients who did not relapse was short in this study, while all those who discontinued JAKi were observed for at least 90 days to determine whether clinical recurrences occurred after discontinuation. Furthermore, the timing of dose reduction and withdrawal was not protocolized, but was based on the subjective judgment of the attending physician, which could lead to potential selection bias in the allocation of patients to the discontinuation or continuation group. This lack of standardized discontinuation timing also makes it difficult to conduct a fair comparison of relapse-free rates between the continuation and discontinuation groups. The sample size was small, and few studies have been conducted on the effects of JAKi discontinuation. However, patients who discontinued JAKi were strictly collected because clinical outcomes for only patients with PRO2 of 0 were evaluated which was “complete remission.” Moreover, the criteria for discontinuation in this study were based on CR, and endoscopic mucosal or histological remission was not confirmed, which may limit the generalizability of our findings. Alternatively, FIT was conducted during JAKi discontinuation. This test is a simple, noninvasive fecal biomarker that is useful for predicting endoscopic severity and clinical outcomes, including fecal calprotectin.^22^ We confirmed that FIT was negative in most patients of the discontinue group. Future studies should investigate whether endoscopic mucosal remission or histological remission could determine the feasibility of JAKi discontinuation.

In conclusion, the relapse rate may increase with JAKi discontinuation and continued treatment is recommended whenever possible, even if CR is obtained. Larger prospective studies with predefined protocols for dose reduction and withdrawal as well as longer observation periods are needed to validate our findings and provide more robust evidence to guide clinical decision-making regarding JAKi discontinuation in patients with UC.

## Supplementary Material

otaf020_suppl_Supplementary_Figures

## Data Availability

The data for this study are available from the corresponding author upon reasonable request.
