# Supplementary figures and images for: Clinical Outcomes for Patients With Ulcerative Colitis in Cases of Withdrawal and Resumption of Janus Kinase Inhibitors: Multicenter Cohort Study
Source: Crohns Colitis 360. 2025 Mar 22;7(2):otaf020. doi: 10.1093/crocol/otaf020 (PMC11995396; doi:10.1093/crocol/otaf020)

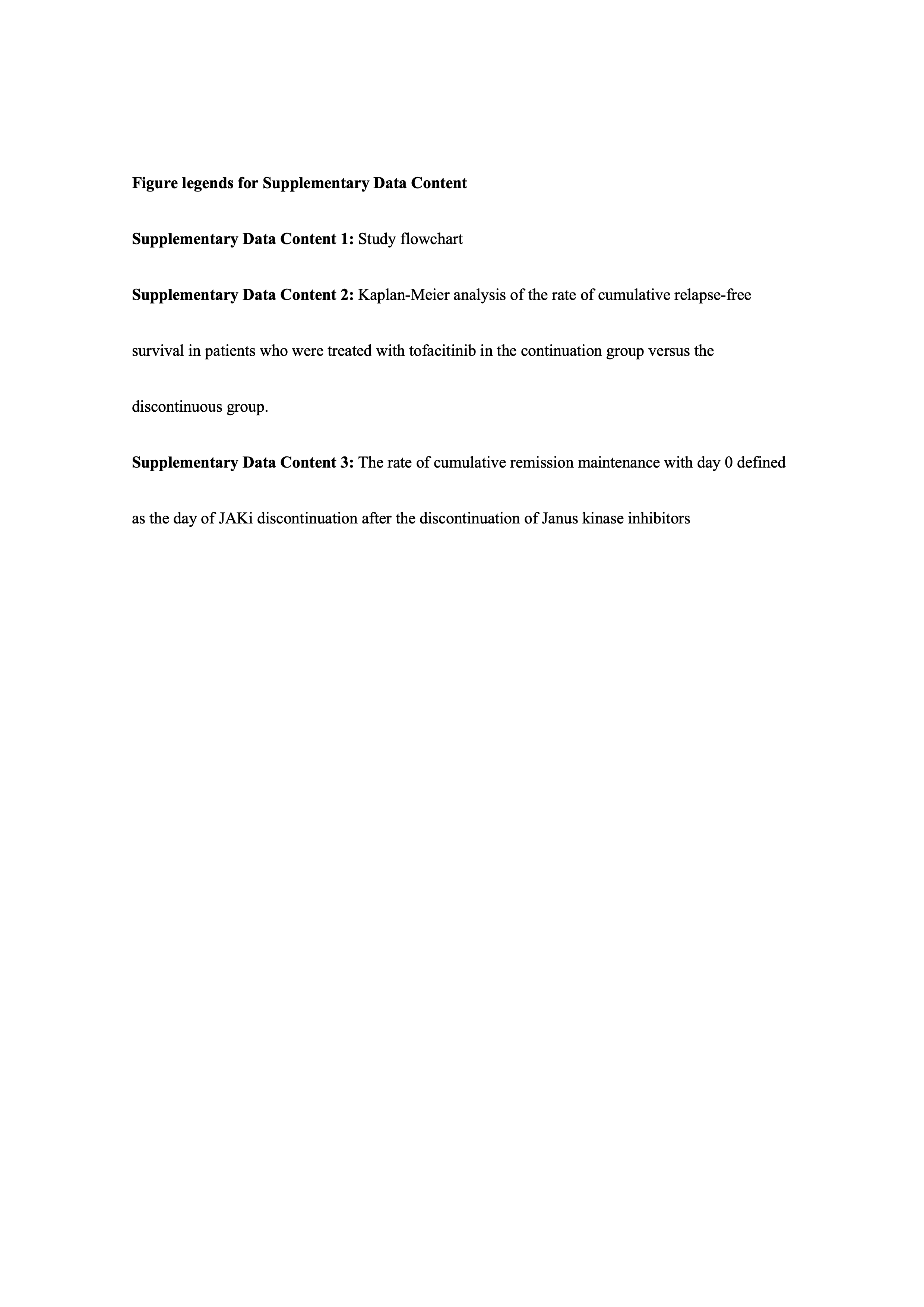

Supplement: otaf020_suppl_Supplementary_Figures [file otaf020_suppl_supplementary_figures.zip › Supplementary Data Contents/Figure legends for Supplementary Data Content.tiff]

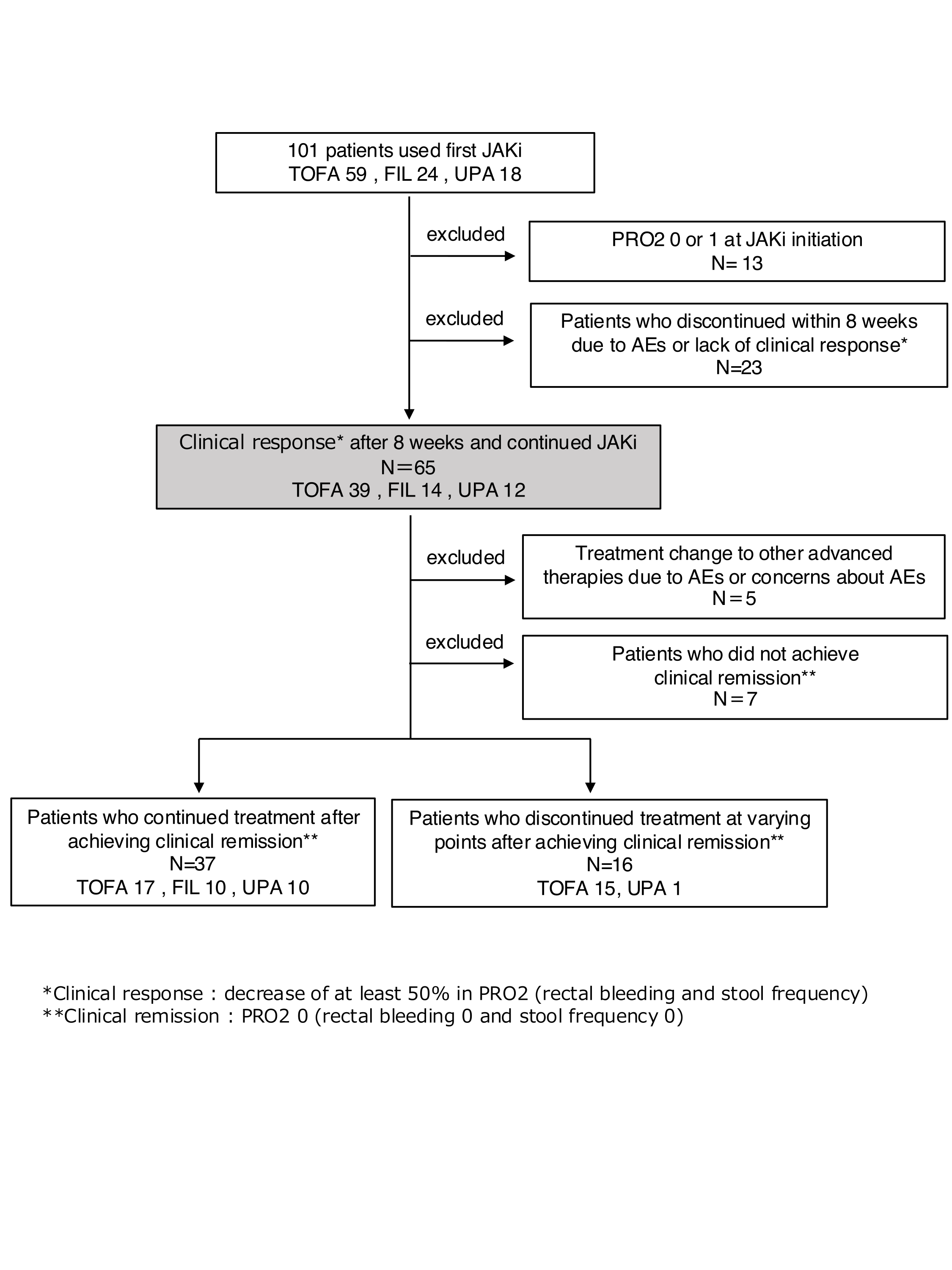

Supplement: otaf020_suppl_Supplementary_Figures [file otaf020_suppl_supplementary_figures.zip › Supplementary Data Contents/Supplementary Data Content 1.tiff]

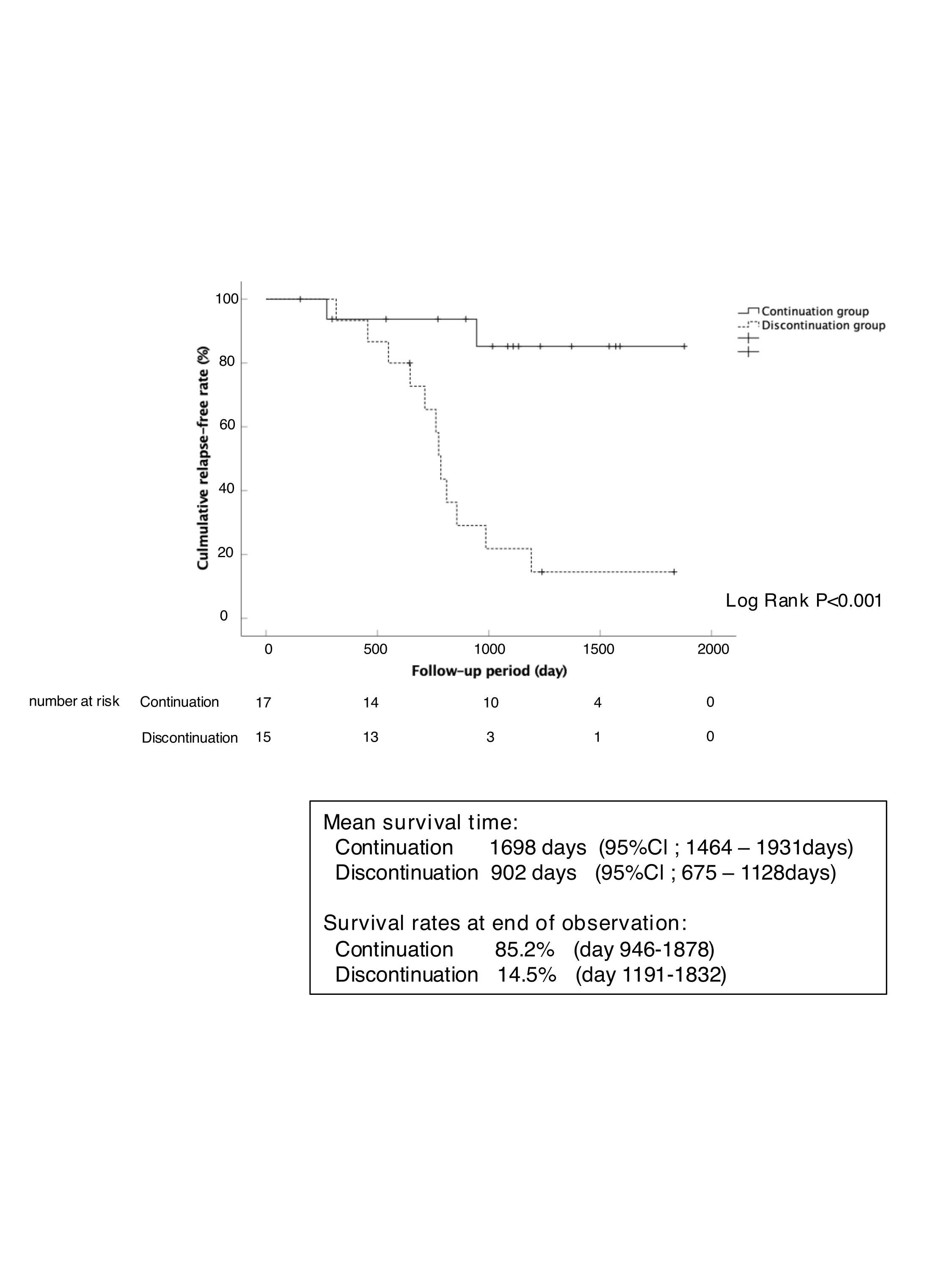

Supplement: otaf020_suppl_Supplementary_Figures [file otaf020_suppl_supplementary_figures.zip › Supplementary Data Contents/Supplementary Data Content 2.tiff]

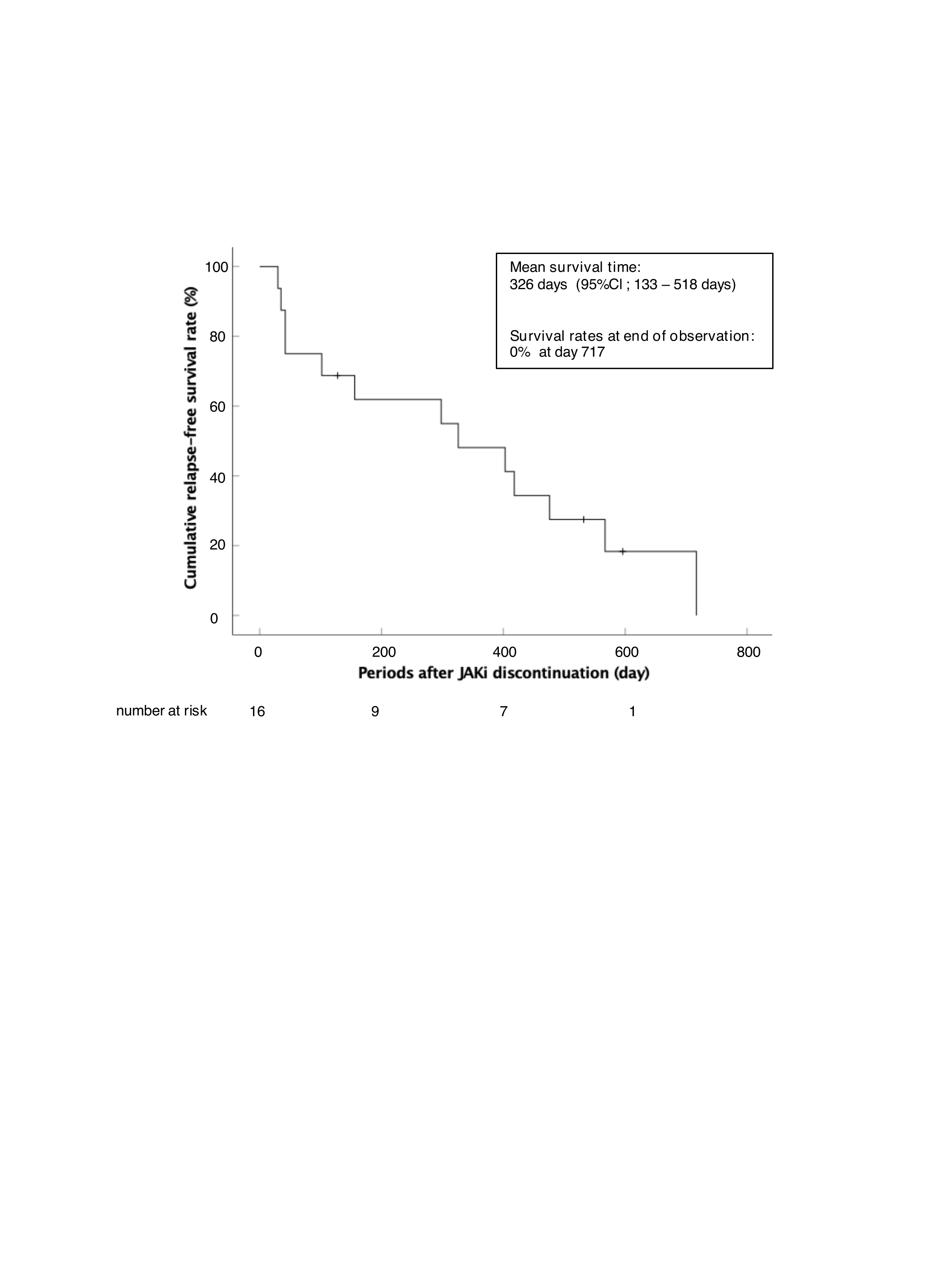

Supplement: otaf020_suppl_Supplementary_Figures [file otaf020_suppl_supplementary_figures.zip › Supplementary Data Contents/Supplementary Data Content 3.tiff]

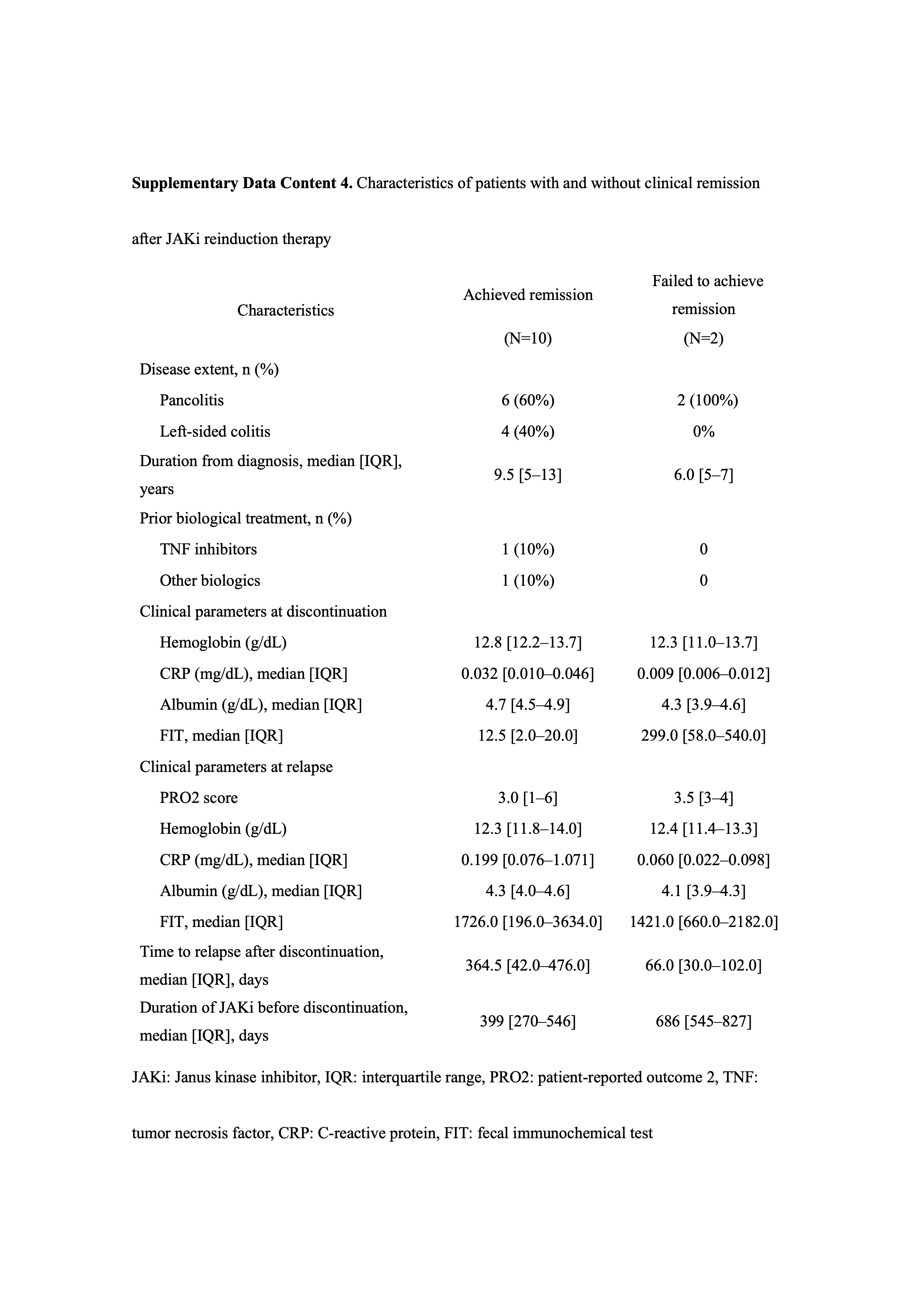

Supplement: otaf020_suppl_Supplementary_Figures [file otaf020_suppl_supplementary_figures.zip › Supplementary Data Contents/Supplementary Data Content 4.tiff]
